# Transcriptome data of *Prorocentrum donghaiense* Lu under nitrogen and phosphorus limitation

**DOI:** 10.1016/j.dib.2018.03.091

**Published:** 2018-03-26

**Authors:** Jing-Jing Li, Wei Xia, Hong-Po Dong, Lin-Jian Ou

**Affiliations:** a Key Laboratory of Eutrophication and Red Tide Prevention of Guangdong Higher Education Institutes, College of Life Science, Jinan University, Guangzhou 510632, China; b School of Ocean and Meteorology, Guangdong Ocean University, Zhanjiang 524088, China

## Abstract

*Prorocentrum donghaiense* Lu is one of the most frequently occurred harmful algae in the coastal waters of China. The growth of *P. donghaiense* can be limited by nitrogen or phosphorus in marine environment. However, molecular mechanism of *P. donghaiense* in response to nitrogen and phosphorus limitation is poorly understood. In this study, we summarized the transcriptome datasets of *P. donghaiense* in response to nitrogen or phosphorus depletion. Raw data of approximately 19 GB in size were generated from IlluminaHiSeq^TM^ 4000 sequencer. From 250, 539, 604 raw reads, 211, 394, 052 clean reads were obtained. The raw data were deposited into SRA database with the BioProject ID 436946. Our dataset will provide more scientific and valuable information for analyses of gene expression related to metabolic processes in *P. donghaiense*.

**Specifications table**

**Table:** 

Subject area	Biology
More specific subject area	Transcriptomics
Type of data	Transcriptome sequences
How data was acquired	IlluminaHiSeq^TM^ 4000
Data format	Raw (FASTQ)
Experimental factors	Monoculture grown in nutrient replete, nitrogen free or phosphorus free media
Experimental features	Comparative transcriptomic analyses of *P. donghaiense* cells grown in different nutrient media
Data source location	Jinan University, China (23˚7'40.8” N 113˚20'56.0”E)
Data accessibility	Data can be accessed from NCBI SRA (Bioproject ID: PRJNA 436946) (https://www.ncbi.nlm.nih.gov/bioproject/PRJNA436946)

**Value of the data**

• The data show changes in gene expression levels of *P. donghaiense* in response to nitrogen or phosphorus limitation, which are valuable for estimating the impact of variation of nutrients on *P. donghaiense* cells.
• The data can be used by other teams studying on the molecular biology of *P. donghaiense*.
• The data will be helpful for analyses of gene expression related to metabolic processes in *P. donghaiense*.

## 1 Data

This article reports the transcriptome data of *Prorocentrum donghaiense* under nutrient replete, nitrogen-limited or phosphorus-limited conditions. The raw data were deposited in the NCBI SRA database as detailed in Table 1.

**Table 1:** SRA accession links for raw transcriptome data of *Prorocentrum donghaiense* grown in nitrogen limitation (NL), phosphorus limitation (PL) or nutrient replete (SL) condition.

**Sample names**	**Biological replicates**	**Accession numbers**	**Accession links**
NL	NL-1	SRX3766193	https://www.ncbi.nlm.nih.gov/sra/?term=SRX3766193
	NL-2	SRX3766192	https://www.ncbi.nlm.nih.gov/sra/?term=SRX3766192
PL	PL-1	SRX3766195	https://www.ncbi.nlm.nih.gov/sra/?term=SRX3766195
	PL-2	SRX3766194	https://www.ncbi.nlm.nih.gov/sra/?term=SRX3766194
SL	SL-1	SRX3766197	https://www.ncbi.nlm.nih.gov/sra/?term=SRX3766197
	SL-2	SRX3766196	https://www.ncbi.nlm.nih.gov/sra/?term=SRX3766196

## 2 Experimental design, materials and methods

### 2.1 Algal strain and experimental design

*P. donghaiense* (No. MEL203) cells were originally isolated from coastal waters of Pearl River Estuary (23°7'40.8” N 113°20'56.0”E), China in 2009, and were preserved in the Research Center of Harmful Algae and Marine Biology in Jinan University, China. For the formal experiments, cultures of *P. donghaiens*e during exponential growth phase were centrifuged at 1400 g for 5 min, and the resultant pellets were transferred into nutrient-replete (882 μmol L^−1^ NO_3_^−^ and 36 μmol L^−1^ PO_4_^3−^), N-free (36 μmol L^−1^ PO_4_^3−^ without NO_3_^-^ addition), and P-free media (882 μmol L^−1^ NO_3_^−^ without PO_4_^3-^ addition), respectively. The cultures were maintained at 20 ± 1 °C in a light-dark cycle of 12 h: 12 h. Cultures were harvested at 12 h after inoculation, and pellets were covered with RNAlater solution (Sigma) and stored at -80°C for further analysis.

### 2.2 Total RNA extraction and quality control

Total RNA was prepared from frozen cells of *P. donghaiense* using the total RNA extraction kit (Magen, shanghai, China). To remove any traces of genomic DNA from RNA extractions, the RNA was treated with RNase-free Dnase (Magen, shanghai, China) according to the manufacture. For quality control, Drop spectrophotometer (Kai Ao, China), Qubit® 3.0 Flurometer (Life Technologies, USA) and Agilent 2100 RNA Nano 6000 Assay Kit (Agilent Technologies, USA) were used to determine the quality, quantity and integrity of the total RNA.

### 2.3 Library preparation and RNA seq

The mRNA was purified from total RNA using poly-T oligo-attached magnetic beads. The purified mRNA was cut into fragments using divalent cations under high temperature. These RNA fragments were generated into first strand cDNA using random hexamer primer and RNase H. After that, the second strand of cDNA was subsequently synthesized using the first strand buffer, dNTPs, DNA polymerase I and RNase H. The cDNA fragments were purified with QiaQuick PCR kits and washed with EB buffer. And then, these fragments were terminally repaired, and poly(A)-tails and adapters were added. The aimed products were separated by agarose gel electrophoresis, and the fragments were PCR amplificated to create a cDNA library. The clustering of the index-coded samples was performed on a cBot cluster generation system using HiSeq PE Cluster Kit v4-cBot-HS (Illumina) and then the library preparations were sequenced using an IlluminaHiSeq^TM^ 4000 sequencer (Illumina, Shanghai, China) and 150 bp paired-end reads were generated.

### 2.4 Transcriptome de novo assembly

In order to get high-quality reads, raw data were processed with Perl scripts to get rid of reads with adaptor sequence, low-quality reads and reads with number of N accounting for more than 5%. High-quality reads were assemblied by Trinity software (version 20140710) [1] (Table 2). The clean data were mapped to the assembled transcript by Bowtie2 to post assembly evaluation [3].

**Table 2:** Statistics of sequence reads under nitrogen limitation (NL), phosphorus limitation (PL) or nutrient replete (SL) conditions.

**Samples**	**Pre-filter**		**Post-filter**	
	**Number of reads**	**Number of bases (bp)**	**Number of reads**	**Number of bases (bp)**
**NL**	80,098,730	10,012,341,250	67,834,274	8,479,284,250
**PL**	83,470,658	10,433,832,250	70,370,816	8,796,352,000
**SL**	86,970,216	10,871,277,000	73,188,962	9,148,620,250

### 2.5 Bioinformatic analyses

**Fig. 1:**
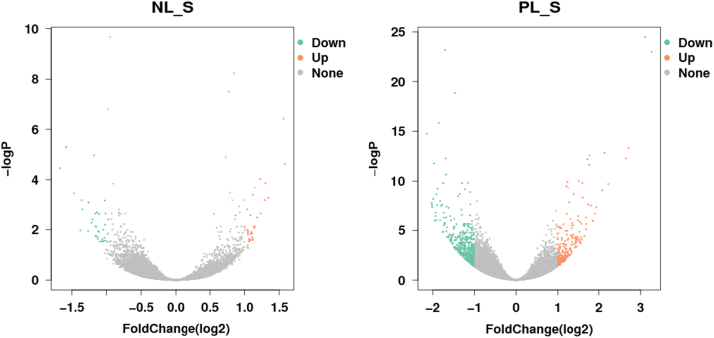
The volcano-plots of differently expressed genes. Note, NL, PL and SL represented cultures under nitrogen limitation, phosphorus limitation and nutrient replete conditions, respectively.

The number of Reads was counted by HTSeq v0.6.0. RPKM (reads per kilobase per million fragments mapped) was then used to quantitatively estimate gene expression values in each sample [2]. DEGseq was used to compare genes that were up-regulated and down-regulated between two samples using a model based on the negative binomial distribution. The P-value could be assigned to each gene and adjusted following the Benjamini and Hochberg's correction for controlling the false discovery rate (FDR). Genes with FDR ≤0.05 and |log_2__ratio|≥2 were identified as differentially expressed genes (DEGs) [3]. When N-limited cultures were compared with N-replete cultures, 34 transcripts were up-regulated and 31 transcripts were down-regulated; Compared between those under phosphorus limitation and nutrient replete conditions, 224 transcripts were up-regulated and 507 transcripts were down-regulated (Fig. 1).
